# Multimodality MRI radiomics analysis of TP53 mutations in triple negative breast cancer

**DOI:** 10.3389/fonc.2023.1153261

**Published:** 2023-03-29

**Authors:** Kun Sun, Hong Zhu, Weimin Chai, Fuhua Yan

**Affiliations:** Department of Radiology, Ruijin Hospital, Shanghai Jiaotong University School of Medicine, Shanghai, China

**Keywords:** radiomics, magnetic resonance imaging, machine learning, TNBC (triple negative breast cancer, support vector machine

## Abstract

**Objectives:**

To explore the value of T1-weighted imaging (T1WI), T2-weighted imaging (T2WI) and diffusion-weighted imaging (DWI) radiomics features reflecting TP53 mutations in patients with triple negative breast cancer (TNBC).

**Study design:**

This retrospective study enrolled 91 patients with TNBC with TP53 testing (64 patients in the training cohort and 27 patients in the validation cohort). A total of 2832 radiomics features were extracted from the first phase of dynamic contrast-enhanced T1WI, T2WI and ADC maps. Analysis of variance (ANOVA) and the Kruskal-Wallis-test were used for feature selection. Then, linear discriminant analysis (LDA), multilayer perceptron (MLP), logistic regression (LR), LR with LASSO, decision tree (DT), naïve Bayes (NB), random forest (RF), and support vector machine (SVM) models were used for classification.

**Results:**

The validation AUCs of the eight classifiers ranged from 0.74 (NB) to 0.85 (SVM). SVM attained the highest AUC (0.85) and diagnostic accuracy (0.82) of all tested models. The top 3 ranking features in the SVM model were T1-square-first order-skewness (coefficient: 1.735), T2-wavelet-LHH-GLCM-joint energy, and T2-wavelet-LHH-GLCM-inverse difference moment (coefficient: -0.654, -0.634).

**Conclusions:**

Radiomics-based analysis with the SVM model is recommended for the detection of TP53 mutations in TNBC. Furthermore, T1WI- and T2WI-related features could be used as noninvasive biomarkers for predicting TP53 mutations.

## Introduction

Triple-negative breast cancer (TNBC) is a molecular subtype of breast cancer defined by a lack of expression of hormonal and human epidermal growth factor receptor-2 (HER2) receptors (1–3). TNBC accounts for 15% of all invasive breast cancers and has a worse prognosis than non-TNBC (4).

TP53 gene mutations are shown to be associated with breast cancer (5). Moreover, breast cancer with TP53 mutations is more likely to be aggressive and resistant to chemotherapy and radiotherapy (5–7). A noninvasive method to detect TP53 mutations in TNBC might optimize treatment plans and improve the prognosis of TNBC patients.

Radiomics analysis has been widely used in cancer detection, diagnosis and prognosis (8, 9). Previous studies (10–12). have shown that radiomics analysis can be used to detect TP53 mutations in cancers. Radiomics analysis combined with different machine learning classifiers may improve the detection of TP53 mutations in TNBC.

Hence, the objective of this study was to explore the potential of radiomics features from T1WI, T2WI and ADC maps to reflect the TP53 status of TNBC and to propose the best classifier for preoperatively assessing TP53 mutations.

## Materials and methods

### Study population

This study is a retrospective subanalysis of data acquired from a prospective study. Institutional review board approval and written informed consent from patients were obtained. A total of 91 patients (mean age, 52 years; age range, 21-77 years) who underwent preoperative MRI, were histopathologically confirmed to have breast cancer and underwent TP53 gene expression profiling between January 2021 and March 2022 were included in this study.

### MR scanning

All MRI examinations were performed using a 1.5 T MRI scanner (MAGNETOM Aera; Siemens Healthcare, Erlangen, Germany) with a dedicated 16-channel bilateral breast coil. The parameters of T2-weighted fast spin-echo imaging are shown in **Appendix A**. Dynamic contrast enhanced (DCE) T1WI was obtained using a fat-suppressed T1-weighted gradient-echo sequence before and four times continuously after the injection of gadolinium contrast medium (Magnevist, Bayer HealthCare Pharmaceuticals Inc., Wayne, New Jersey, USA), which was administered intravenously by a power injector at a dose of 0.1 mmol/kg body weight at a rate of 2.5 mL/s, followed by a 20-mL saline flush at the same injection rate. The scan parameters of DCE and DWI are in **Appendix B**.

### Image postprocessing and lesion segmentation

All the data were assessed by SK and CWM (with 9 years and 13 years of experience in breast imaging, respectively) to identify all lesions by using T2-weighted images (T2WI), precontrast T1-weighted images (T1WI) and the first phase of postcontrast T1WI. Specifically, the clinical information and X-ray and US images were provided to the radiologists. The lesions were then manually segmented in the subtraction images of postcontrast T1WI and precontrast T1WI on all visible sections, T2WI, and ADC maps, resulting in a three-dimensional image of the lesion. The lesions were further segmented by using the inner border of the lesion to minimize partial volume effects. Note that all volumes of interests (VOIs) were manually segmented and labeled *via* a free open-source software package (3D slicer, version 3.4.0).

### Feature extraction

A total of 2832 radiomics features were obtained from each segmented lesion, which were categorized into the following nine groups: (1) 14 shape features, (2) 19 first-order features, (3) 24 gray level co-occurrence matrix (GLCM) features, (4) 14 gray level dependence matrix (GLDM) features, (5) 16 gray level run length matrix (GLRLM) features, (6) 16 gray level size zone matrix (GLSZM) features, (7) 5 neighbouring gray tone difference matrix (NGTDM) features, (8) 93 square-related features, and (9) 744 wavelet related features. The details of the extracted radiomics features are shown in **Appendix C**.

### Feature selection

We selected 64 cases as the training dataset (46/18 = positive/negative). We also selected another 27 cases as the independent testing dataset (20/7 = positive/negative).

To remove the imbalance of the training dataset, we upsampled the data by repeating random cases to balance positive/negative samples. We applied normalization to the feature matrix. Each feature vector was subtracted by the mean value of the vector and divided by its length. Since the dimension of the feature space was high, we compared the similarity of each feature pair. If the Pearson correlation coefficient of the feature pair was greater than 0.9, which means that the two features are the same, then we removed one of them. After this process, the dimension of the feature space was reduced, and each feature was independent of the others. Before building the model, we used analysis of variance (ANOVA), and recursive feature elimination (RFE) to select features. ANOVA is a common method to explore the significant features corresponding to the labels. The F value was calculated to evaluate the relationship between features and the label. We sorted features according to the corresponding F value and selected a specific number of features to build the model. The goal of RFE is to select features based on a classifier.

### Machine learning classification

We used linear discriminant analysis (LDA), multilayer perceptron (MLP), logistic regression (LR), LR with LASSO, decision tree (DT), naïve Bayes (NB), random forest (RF), and support vector machine (SVM) models. LDA is a linear classifier that fits class conditional densities to the data and uses the Bayes rule. MLP analysis is based on a neural network with multi-hidden layers and is used to find the mapping from inputted features to the label. Here, we used 1 hidden layer with 100 hidden units. The nonlinear activation function was a rectified linear unit function, and the optimizer was Adam with step 0. 001. LR is a linear classifier that combines all of the features. LR with LASSO constraint is a linear classifier based on logistic regression. The L1 norm is added in the final loss function, and the weights are constrained, which makes the features sparse. The DT method is a nonparametric supervised learning method that can be used for classification with high interpretation and is a kind of probabilistic classifier based on Bayes theorem. The NB method requires the number of parameters to be linear in the number of features. The RF is an ensemble learning method that combines multiple decision trees at different subsets of the training dataset. RF is an effective method to avoid overfitting. SVM was an effective and robust classifier to build the model. The kernel function has the ability to map the features into a higher dimension to search the hyperplane for separating the cases with different labels. Here, we used the linear kernel function because it better explained the coefficients of the features for the final model. To determine the hyperparameter (e.g., the number of features) of the model, we applied 10-fold cross validation on the training dataset. The hyperparameters were set according to the models’ performance on the validation dataset.

### Statistical analysis

**Figure 1:**
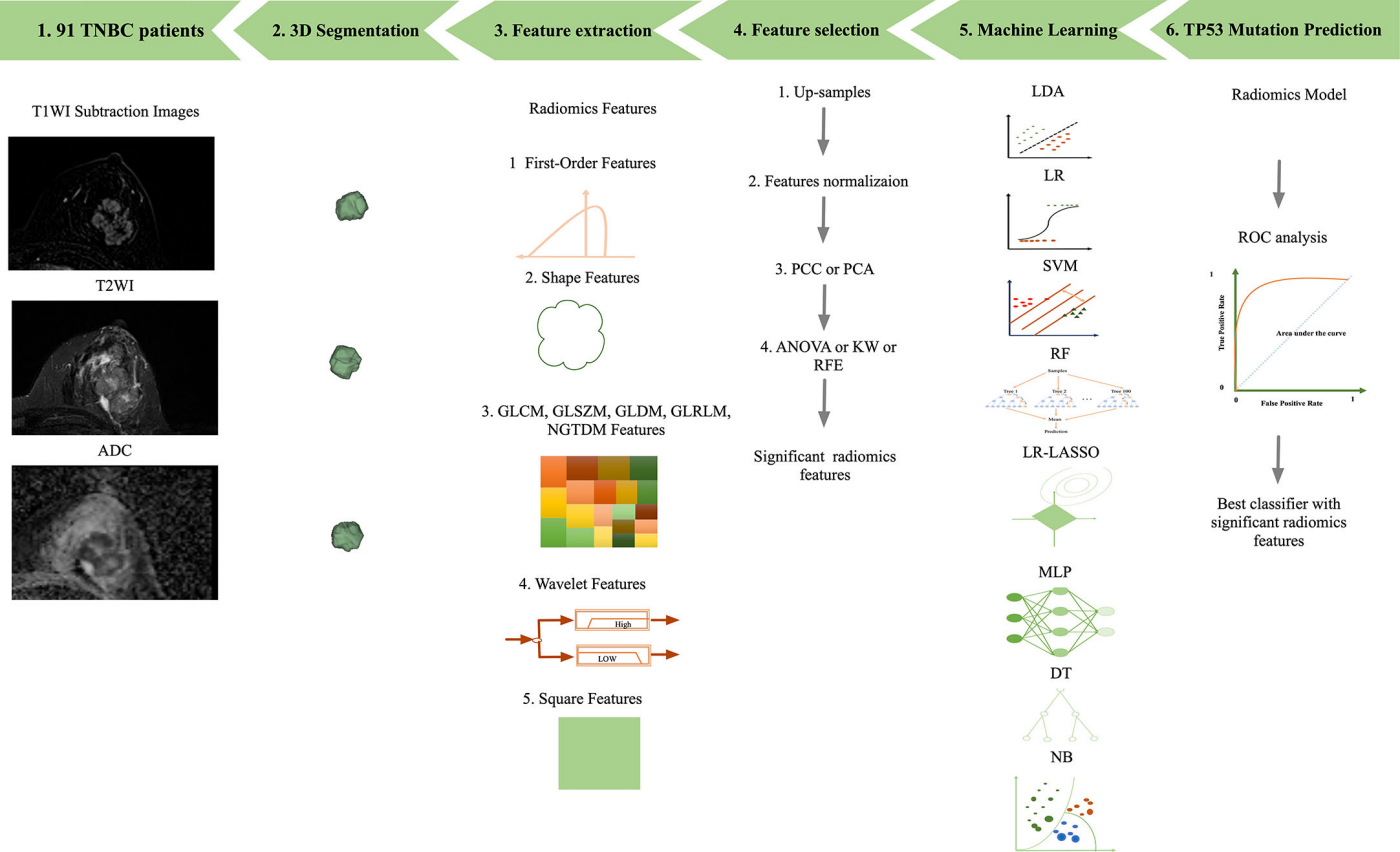
Workflow of image processing. 1) T1WI, T2WI, and ADC data of 91 breast cancer patients. 2) 3D segmentations of lesions shown as surface-shaded 3D renderings. 3) Extraction of radiomic features, i.e., first-order, shape, texture features, square and wavelet features 4) feature selection. 5) Eight machine learning classifications were all performed. 6) ROC analysis was used to assess the diagnostic performance of the radiomics model in the detection of TP53 mutations in TNBC.

Continuous variables are expressed as the mean value ± standard deviation (SD). The performance of the model was evaluated using receiver operating characteristic (ROC) curve analysis. The area under the ROC curve (AUC) was calculated for quantification. The accuracy, sensitivity, specificity, positive predictive value (PPV), and negative predictive value (NPV) were also calculated at a cutoff value that maximized the value of the Youden index. We also estimated the 95% confidence interval by bootstrapping with 1000 samples. All of the processes were implemented with FeAture Explorer Pro (FAEPro, V 0.4.4) (13) in Python (3.7.6) or with SPSS version 23.0 software. *A P* value less than 0.05 was regarded as a significant difference. An overview of the workflow used in this study is illustrated in **Figure 1**.

## Results

### Clinical characteristics

There were no significant differences in demographic characteristics between TNBC patients with TP53 mutation (mean age, 51.2 ± 12.4 years) and TNBC patients without TP53 mutation (mean age, 54.9 ± 13.3 years; *p* = 0.215). Details are shown in **Table 1**.

**Table 1:** The clinico-pathological features of TNBC patients with TP53 mutations and without TP53 mutations.

Characteristic	TP53 mutations	Without TP53 mutations	*p*
Clinical features			
Mean age	51.2 ± 12.4	54.9 ± 13.3	0.215
Menstrual status			0.801
Premenopausal	31/66 (47%)	11//25 (44%)	
Postmenopausal	35/66(53%)	14/25 (56%)	
T stage of MRI			0.657
<=1	0/66 (0)	1/25 (4%)	
1-2	24/66 (36%)	9/25 (36%)	
> 2	42/66 (64%)	15/25 (60%)	
MRI-BI-RADS			0.105
4	27/66 (41%)	15/25 (60%)	
5	39/66 (59%)	10/25 (40%)	
Pathological features			
Lymphovascular invasion	10/66 (15%)	5/25 (20%)	0.580
Lymph nodes metastasis	20/66 (30%)	6/38 (16%)	0.555

### Pathological features

**Figure 2:**
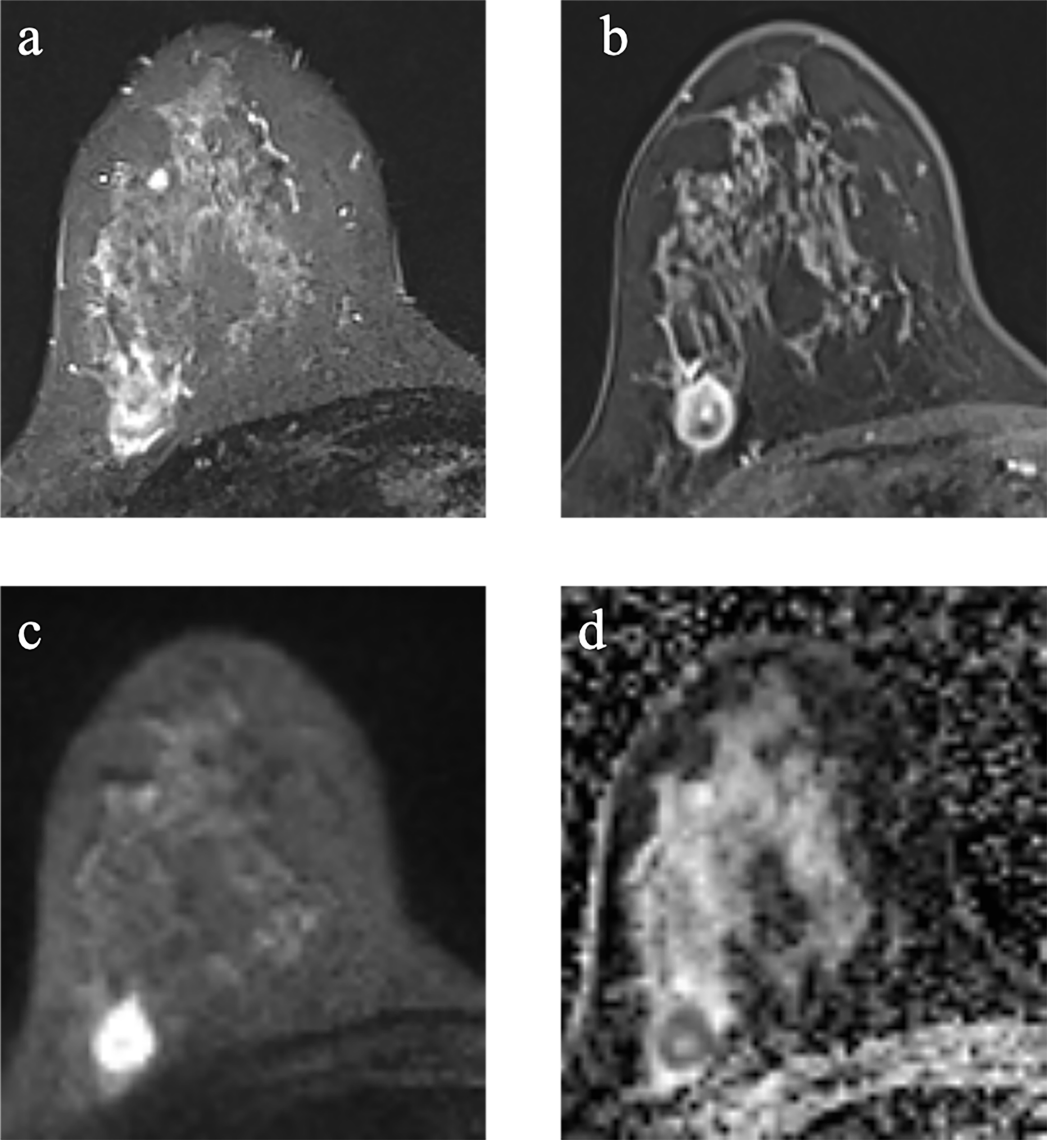
Example images of a 29-year-old woman with TNBC with TP53 mutation in the right breast **(A-D)**. **(A)**: T2-weighted-image. **(B)**: the first phase of T1WI after contrast enhancement. **(C)**: diffusion-weighted image of b_1000_. **(D)**: apparent diffusion coefficient (ADC) map.

**Figure 3:**
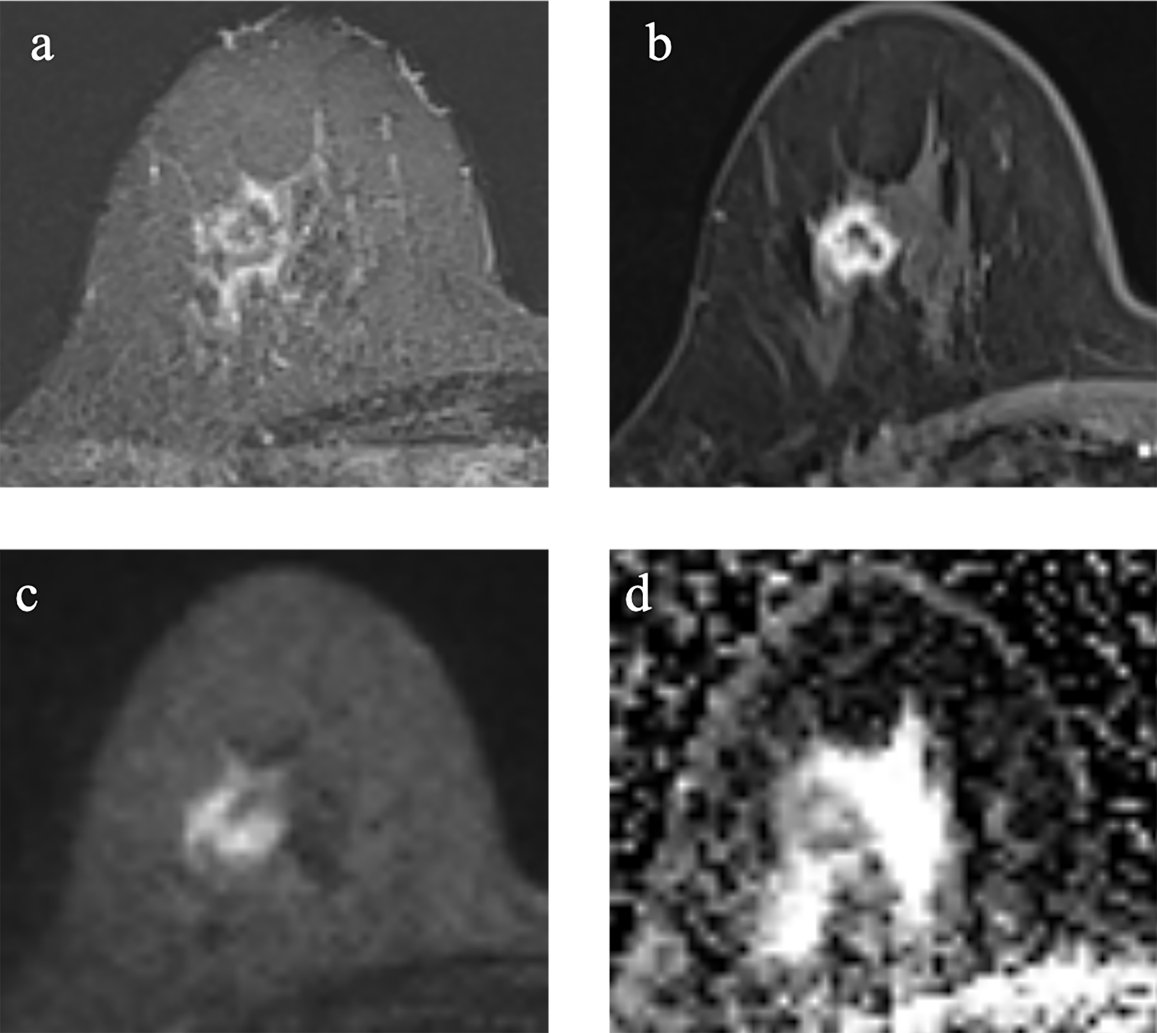
Example images of a 70-year-old woman with TNBC without TP53 mutation in the right breast. **(A-D)**. **(A)**: T2-weighted-image. **(B)**: the first phase of T1WI after contrast enhancement. **(C)**: diffusion-weighted image of b_1000_. **(D)**: apparent diffusion coefficient (ADC) map.

Of the 91 TNBC lesions, 66 lesions with TP53 mutations, and other 25 lesions without TP53 mutations. Cases of TNBC with and without TP53 mutations are shown in **Figures 2**, **3**.

### Performance of the LDA, MLP, LR, LR with LASSO, DT, NB, RF, and SVM models for the training and testing cohorts

The predictive performance of the LDA, MLP, LR, LR with LASSO, DT, NB, RF and SVM models are shown in **Table 2**. For the LR and LDA classifiers, only 1 feature was selected for predicting TP53 mutation in TNBC. The accuracy and area under the curve (AUC) of the LR classifier for TP53 mutations in the training cohort were 72%, and 0.72, respectively, while those in the testing cohort were 85%, and 0.75, respectively. The accuracy and AUC of the LDA classifier for TP53 mutations in the training cohort were 72%, and 0.72, respectively, while those in the testing cohort were 85%, and 0.75, respectively. The selected feature in the LR and LDA models was T2-wavelet-LHH-GLCM-JointEnergy (coefficient: -5.119, -4.486).

**Table 2:** Predictive performance for LDA, RF, SVM, LR, LR-LASSO, MLP, DT, and NB in the training and testing cohorts.

Models	Training cohort						Testing cohort					
	AUC	ACC	SEN	SPE	PPV	NPV	AUC	ACC	SEN	SPE	PPV	NPV
LDA	0.72	72%	76%	61%	83%	50%	0.75	85%	100%	43%	83%	100%
RF	1.0	100%	100%	100%	100%	100%	0.83	74%	65%	100%	100%	50%
SVM	0.75	73%	78%	61%	84%	52%	0.85	82%	80%	86%	94%	60%
LR	0.72	72%	76%	61%	83%	50%	0.75	85%	100%	43%	83%	100%
LR-LASSO	0.76	56%	39%	100%	100%	39%	0.76	85%	100%	43%	83%	100%
MLP	0.72	75%	80%	61%	84%	55%	0.85	70%	60%	100%	100%	47%
DT	1.0	100%	100%	100%	100%	100%	0.76	78%	80%	71%	89%	56%
NB	0.71	72%	76%	61%	83%	50%	0.74	70%	70%	71%	88%	45%

For the RF classifier, 19 features were selected for predicting TP53 mutation in TNBC. The accuracy and AUC for TP53 mutations in the training cohort were 100%, and 1.00, respectively, while those in the testing cohort were 74%, and 0.83, respectively. The selected features in the RF classifier are shown in the **Appendix D**.

**Figure 4:**
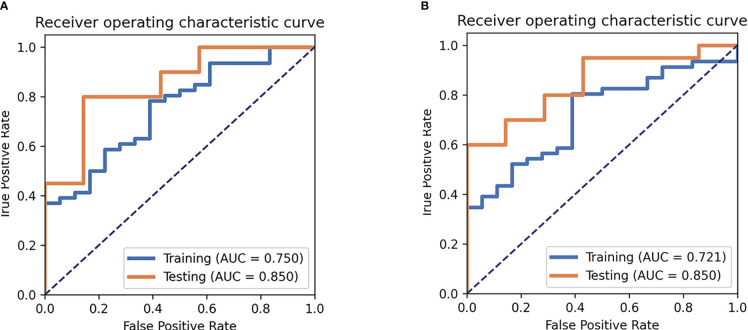
Receiver operating characteristic curve (ROC) analysis for the SVM **(A)** and MLP **(B)** model.

**Figure 5:**
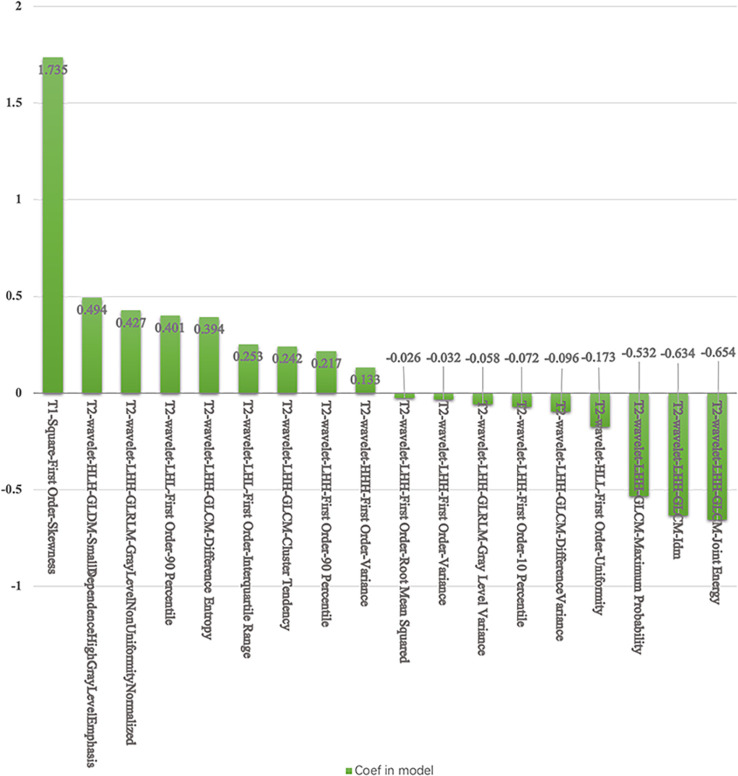
The selected features and coefficients in the SVM.

For the SVM classifier, 18 features were selected for predicting TP53 mutation in TNBC. The accuracy and AUC for TP53 mutations in the training cohort were 73%, and 0.75, respectively, while those in the testing cohort were 82%, and 0.85, respectively. The ROC curves are shown in **Figure 4**. The selected features in the SVM classifier are shown in the **Figure 5**.

For the MLP classifier, 16 features were selected for predicting TP53 mutation in TNBC. The accuracy and AUC for TP53 mutations in the training cohort were 75%, and 0.72, respectively, while those in the testing cohort were 70%, and 0.85, respectively. The ROC curves are shown in **Figure 4**. The selected features in the MLP classifier are shown in the **Appendix E**.

For the LR with LASSO classifier, 3 features were selected for predicting TP53 mutation in TNBC. The accuracy and AUC for TP53 mutations in the training cohort were 56%, and 0.76, respectively, while those in the testing cohort were 85%, and 0.76, respectively. The selected features in the LR with LASSO classifier are shown in the **Appendix F**.

For the DT classifier, 18 features were selected for predicting TP53 mutation in TNBC. The accuracy and AUC for TP53 mutations in the training cohort were 100%, and 1.0, respectively, while those in the testing cohort were 78%, and 0.76, respectively. The selected features in the DT classifier are shown in the **Appendix G**.

For the NB classifier, 18 features were selected for predicting TP53 mutation in TNBC. The accuracy and AUC for TP53 mutations in the training cohort were 72%, and, 0.71 respectively, while those in the testing cohort were 70%, and 0.74, respectively. The selected features in the NB classifier are shown in the **Appendix H**.

## Discussion

In our study, we investigated the value of radiomics features based on T2WI, T1WI, and ADC maps in identifying TP53 mutations in triple negative breast cancer (TNBC). Our results revealed that radiomics features combined with SVM could achieve a better predictive performance than other models for TP53 mutations in TNBC, with an AUC of 0.85. Furthermore, T1WI-square features and T2WI-wavelet features with higher coefficients were recommended as noninvasive biomarkers for the prediction of TP53 mutations in TNBC.

TP53 somatic mutations are one of the most common genetic abnormalities associated with cancer (5, 14). The value of TP53 mutation status for predicting tumor response to treatment and patient outcome has been evaluated in numerous cancers, especially breast cancer (2). Many of those studies (5, 15, 16) have shown that TP53 mutations are associated with a poorer prognosis. Hence, in this research, we wanted to determine whether multimodality MRI biomarkers are associated with TP53 mutations in TNBC using various machine learning classifiers.

Radiomics analysis has already been used in several studies of TP53 mutations in breast cancer (10, 17). Moon et al. (17) used texture and morphology analysis to evaluate TP53 mutations and found that texture analysis could be used to identify TP53 mutations. However, in their research, all types of breast cancer were included. In our research, we wanted to find the relationship between radiomics features and TP53 mutations in TNBC. In our previous study (10), we only used radiomics features of T1WI images to identify breast cancer with or without TP53 mutations. We found that the clinicopathological-radiomics features combined with SVM model could be used as non-invasive biomarker for the prediction of TP53 mutations. However, in this study, we enrolled multi-modality MRI data, and focused on TNBC with various machine learning classifiers. We wanted to identify the most important feature and the best classifier to predict TP53 mutations in TNBC.

Many machine learning methods can be used for classification (18–21). In this research, we examined eight commonly used classifiers with the goal of identifying the best classifier detect TP53 mutations in TNBC. In our research, the SVM and MLP classifiers achieved the highest AUCs, while the SVM classifier had relatively higher accuracy than the MLP classifier. The SVM classifier (19) has strong generalization power and was easily solves limited sample problems, which makes it outstanding compared to other classifiers. Hence, SVM was recommend for the TP53 mutations in TNBC.

T1WI, T2WI and DWI are the most commonly used MRI sequences for breast cancer diagnosis (22). In our research, T1WI-square features and T2WI-wavelet features were the most important features associated with TP53 mutations in TNBC. Features based on ADC maps were not selected in any of these models, which means that in the high-order feature groups, the value of ADC maps was less than those of T1WI and T2WI. The reason might be that, both TNBC with or without TP53 mutations are associated with central fibrosis and necrosis, the whole tumor analysis of ADCs might not show the differences.

T1-square-first order-skewness, T2-wavelet-LHH-GLCM-joint energy, and T2-wavelet-LHH-GLCM-IDM were the significant selected features in the SVM model for the TP53 mutations in TNBC. T1WI-square-first order-skewness measures the square of asymmetry of the distribution of the mean T1WI intensities, which may correlate more with tumor heterogeneity than the other features. Wavelet transform (23, 24) applies a high- or low-pass filter in all three dimensions in 3D images to obtain eight different wavelet features. Energy is a measure of homogeneous patterns in the T2WI. A greater energy implies that there are more instances of intensity value pairs in the T2WI that neighbor each other at higher frequencies. IDM is a measure of the local homogeneity of T2WI. IDM weights are the inverse of the contrast weights (25). These two GLCM features based on T2WI were related to TP53 mutations in TNBC.

Our study has several limitations. First, our study is a single-center retrospective study. Second, even though we had enrolled all TNBC patients with TP53 testing one year in our hospital. TNBC only accounts for 15% (4), and only limited TNBC with TP53 testing resulting in 91 patients enrolled in our study. The limited sample size of patients may result in data imbalance. Further study with larger sample size is recommended.

In conclusion, radiomics-based analysis with the SVM model is recommended for the detection of TP53 mutations in TNBC. Furthermore, T1WI-square and T2WI-wavelet related features could be used as noninvasive biomarkers for predicting TP53 mutations.

## Data availability statement

The original contributions presented in the study are included in the article/**Supplementary Material**. Further inquiries can be directed to the corresponding author.

## Ethics statement

The studies involving human participants were reviewed and approved by a retrospective subanalysis of data acquired from a prospective study. Institutional review board approval and written informed consent from patients were obtained. The patients/participants provided their written informed consent to participate in this study.

## Author contributions

All authors listed have made a substantial, direct, and intellectual contribution to the work and approved it for publication.
