# Supplementary material for: Multimodality MRI radiomics analysis of TP53 mutations in triple negative breast cancer
Source: Front Oncol. 2023 Mar 29;13:1153261. doi: 10.3389/fonc.2023.1153261 (PMC10090452; doi:10.3389/fonc.2023.1153261)
Supplement: Supplementary file 1 [file DataSheet_1.pdf]

Appendix A

TR/TE of 4990/65 ms, field of view of 360 × 360 mm^2, slice thickness of 4.0 mm, and acquisition time of 2 mins and 11 seconds

Appendix B

The parameters of T1WI: TR/TE of 4.5/1.6 ms, field of view of 340 × 340 mm^2, matrix of 384 × 385, flip angle of 10 degrees, slice thickness of 1.0 mm, and number of slices of 104, with a total duration of T1WI of 7 mins and 30 seconds.

The parameters of DWI: b values of 0 and 1000s/mm^2, TR/TE of 7100/68 ms; field of view of 360 × 256 mm^2, matrix of 172 × 62, number of sections of 30, section thickness of 4mm, number of readout segments of 5, orientation of transverse, flip angle of 180, iPAT mode of GRAPPA, acceleration factor of 2, and acquisition time of 4 min 16 s.

Appendix C

Detailed information about extracted radiomics features

(1) **18 First-order (FO) features**: 10 percentile, 90 percentile, Energy, Entropy, Interquartile Range, Kurtosis, Maximum, Mean Absolute Deviation, Mean, Median, Minimum, Range, Robust Mean Absolute Deviation, Root Mean Squared, Skewness, Total Energy, Uniformity, and Variance.

(2) **14 Shape features**: Elongation, Flatness, Least Axis Length, Major Axis Length, Maximum 2D Diameter Column, Maximum 2D Diameter Row, Maximum 2D Diameter Slice, Maximum 3D Diameter, Mesh Volume, Minor Axis Length, Sphericity, Surface Area, Surface Volume Ratio, and Voxel Volume.

(3) **24 Gray level co-occurrence matrix (GLCM) features**: Autocorrelation, Joint Average, Cluster Prominence, Cluster Shade, Cluster Tendency, Contrast, Correlation, Difference Average, Difference Entropy, Difference Variance, Joint Energy, Joint Entropy, Imc1, Imc2, Idm, Idmn, Id, Idn, Inverse Variance, Maximum Probability, Sum Entropy, and Sum Squares.

(4) **16 Gray level run length matrix (GLRLM) features**: Gray Level Non Uniformity, Gray Level Variance, Gray Level Non Uniformity Normalized, High Gray Level Run Emphasis, Long Run Emphasis, Long Run High Gray Level Emphasis, Long Run Low Gray Level Emphasis, Low Gray Level Run Emphasis, Run Entropy, Run Length Non Uniformity, Run Length Non Uniformity Normalized, Run Percentage, Run Variance, Short Run Emphasis Short Run High Gray Level Emphasis, and Short Run Low Gray Level Emphasis.

(5) **16 Gray level size zone matrix (GLSZM) features**: Gray Level Non Uniformity, Gray Level Non Uniformity Normalized, Gray Level Variance, High Gray Level Zone Emphasis, Large Area Emphasis, Large Area High Gray Level Emphasis, Large Area Low Gray Level Emphasis,

Low Gray Level Zone Emphasis, Size Zone Non Uniformity, Size Zone Non Uniformity Normalized, Small Area Emphasis, Small Area High Gray Level Emphasis, Small Area Low Gray Level Emphasis, Zone Entropy, Zone Percentage, and Zone Variance.

(6) **5 neighboring gray tone difference matrix (NGTDM) features**: Contrast Feature Value, Busyness Feature Value, Complexity Feature Value, and Strength Feature Value.

(7) **14 Gray level dependence matrix (GLDM) features**: Dependence Entropy, Dependence Non Uniformity, Dependence Non Uniformity Normalized, Dependence Variance, Gray Level Non Uniformity, Gray Level Variance, Gray Level Non Uniformity, Gray Level Variance, High Gray Level Emphasis, Large Dependence Emphasis, Large Dependence High Gray Level Emphasis, Large Dependence Low Gray Level Emphasis, Low Gray Level Emphasis, Small Dependence Emphasis, Small Dependence High Gray Level Emphasis, and Small Dependence Low Gray Level Emphasis.

(8) **93 Square-related features**: 18 square-first-order features, 24 square-GLCM features, 16 square-GLRLM features, 16 square-GLSZM features, 5 square-NGTDM features, and 14 square-GLDM features.

(9) **744 Wavelet related features.**

Appendix D

The selected features and the rank in the RF classifier

Features	Features Rank
T2-GLRLM-High Gray Level Run Emphasis	1
T2-wavelet-HHH-First Order-Variance	2
T2-wavelet-HLH-GLDM-Small Dependence High Gray Level Emphasis	3
T2-wavelet-HLL-First Order-Uniformity	4
T2-wavelet-LHH-First Order-10 Percentile	5
T2-wavelet-LHH-First Order-90 Percentile	6
T2-wavelet-LHH-First Order-Root Mean Squared	7
T2-wavelet-LHH-First Order-Variance	8
T2-wavelet-LHH-GLCM-Cluster Tendency	9
T2-wavelet-LHH-GLCM-Difference Entropy	10
T2-wavelet-LHH- GLCM-Difference Variance	11
T2-wavelet-LHH- GLCM-Idm	12
T2-wavelet-LHH- GLCM-Joint Energy	13
T2-wavelet-LHH-GLCM-MaximumProbability	14
T2-wavelet-LHH-GLRLM-Gray Level NonUniformity Normalized	15
T2-wavelet-LHH-GLRLM-Gray Level Variance	16
T2-wavelet-LHL-First Order-90 Percentile	17
T2-wavelet-LHL-First Order-Interquartile Range	18
T1-Square-First Order-Skewness	19

Appendix E

The selected features and the rank in the MLP classifier

Features	Rank
T2-wavelet-HLH-GLDM-Small Dependence High Gray Level Emphasis	1
T2-wavelet-HLL-First Order-Uniformity	2
T2-wavelet-LHH-First Order-10 Percentile	3
T2-wavelet-LHH-First Order-90 Percentile	4
T2-wavelet-LHH-First Order-Root Mean Squared	5
T2-wavelet-LHH-First Order-Variance	6
T2-wavelet-LHH-GLCM-Cluster Tendency	7
T2-wavelet-LHH-GLCM-Difference Entropy	8
T2-wavelet-LHH-GLCM-Difference Variance	9
T2-wavelet-LHH-GLCM-Idm	10
T2-wavelet-LHH-GLCM-Joint Energy	11
T2-wavelet-LHH-GLCM-Maximum Probability	12
T2-wavelet-LHH-GLRLM-Gray Level NonUniformity Normalized	13
T2-wavelet-LHH-GLRLM-Gray Level Variance	14
T2-wavelet-LHL-First Order-90 Percentile	15
T2-wavelet-LHL-First Order-Interquartile Range	16

Appendix F

The selected features and coefficients in the LR with LASSO classifier

Features	Coef in model
T1-Square-First Order-Skewness	1.771
T2-wavelet-LHH-GLCM-Maximum Probability	-1.915
T2-wavelet-LHH-GLCM-Joint Energy	-1.333

Appendix G

The selected features and the rank in the DT classifier

Features	Rank
T2-wavelet-HHH-First Order-Variance	1
T2-wavelet-HLH-GLDM-Small Dependence High Gray Level Emphasis	2
T2-wavelet-HLL-First Order-Uniformity	3
T2-wavelet-LHH-First Order-10 Percentile	4
T2-wavelet-LHH-First Order-90 Percentile	5
T2-wavelet-LHH-First Order-Root Mean Squared	6
T2-wavelet-LHH-First Order-Variance	7
T2-wavelet-LHH-GLCM-Cluster Tendency	8
T2-wavelet-LHH-GLCM-Difference Entropy	9
T2-wavelet-LHH-GLCM-Difference Variance	10
T2-wavelet-LHH-GLCM-Idm	11
T2-wavelet-LHH-GLCM-Joint Energy	12
T2-wavelet-LHH GLCM-Maximum Probability	13
T2-wavelet-LHH-GLCM-Gray Level NonUniformity Normalized	14

T2-wavelet-LHH-GLRLM-Gray Level Variance	15
T2-wavelet-LHL-First Order-90 Percentile	16
T2-wavelet-LHL-First Order-Interquartile Range	17
T1-square-first Order-Skewness	18

Appendix H

The selected features and the rank in the NB classifier

Features	Rank
T2-wavelet-HHH-First Order-Variance	1
T2-wavelet-HLH-GLDM-Small Dependence High Gray Level Emphasis	2
T2-wavelet-HLL-First Order-Uniformity	3
T2-wavelet-LHH-First Order-10 Percentile	4
T2-wavelet-LHH-First Order-90 Percentile	5
T2-wavelet-LHH-First Order-Root Mean Squared	6
T2-wavelet-LHH-First Order-Variance	7
T2-wavelet-LHH-GLCM-Cluster Tendency	8
T2-wavelet-LHH-GLCM-Difference Entropy	9
T2-wavelet-LHH-GLCM-Difference Variance	10
T2-wavelet-LHH-GLCM-Idm	11
T2-wavelet-LHH-GLCM-Joint Energy	12
T2-wavelet-LHH-GLCM-MaximumProbability	13
T2-wavelet-LHH-GLCM-Gray Level NonUniformity Normalized	14
T2-wavelet-LHH-GLRLM-Gray Level Variance	15
T2-wavelet-LHL-First Order-90 Percentile	16
T2-wavelet-LHL-First Order-Interquartile Range	17
T1-square-First Order-Skewness	18
